# Qualitative study exploring the experiences of sexual dysfunction in premenopausal women with type 1 diabetes

**DOI:** 10.1111/dme.15439

**Published:** 2024-09-20

**Authors:** Rahab Hashim, Rita Forde, Judith Parsons, Davide Ausili, Angus Forbes

**Affiliations:** ^1^ Division of Care in Long‐Term Conditions, Florence Nightingale Faculty of Nursing, Midwifery and Palliative Care King's College London London UK; ^2^ University Hospital Bristol and Weston NHS Trust Weston Hospital North Somerset UK; ^3^ School of Nursing and Midwifery University College Cork Cork Ireland; ^4^ Milano‐Bicocca University Milano Italy

**Keywords:** female sexual dysfunction, patient‐reported outcomes, qualitative study, sexual activity, type 1 diabetes

## Abstract

**Aims:**

To explore the sexual experiences and interactions of women with type 1 diabetes to explicate an understanding of the impact of diabetes on women's sexual function. The study was conducted as part of a wider project to develop a patient‐reported outcome measure to assess sexual dysfunction (SD) in premenopausal women with type 1 diabetes.

**Methods:**

A qualitative study using face‐to‐face and virtual semi‐structured interviews was conducted with premenopausal women with type 1 diabetes who have had some difficulties related to sexual functioning. Participants were recruited from two National Health Services (NHS) sites in the UK and from social media platforms. The data were analysed to generate themes using Framework Analysis approach.

**Results:**

Eighteen women, aged 22–49, were interviewed (NHS sites *n* = 13; online *n* = 5). Five themes related to women experiences of SD were identified, these were; initiation of sexual activity, sexual confidence, sexual enjoyment, sexual engagement and sexual desire.

**Conclusions:**

SD in women with type 1 diabetes is a complex phenomenon impacting their experiences and quality of life. SD is related to multiple interacting biopsychosocial factors related to diabetes, including blood glucose levels, diabetes treatments, technologies and complications. A targeted measure of SD for women with type 1 diabetes specifically would allow for these factors to be assessed routinely in clinical care.

## Graphical abstract

Graphical abstract outlining FSD themes in type 1 diabetes.

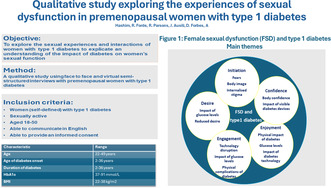

**What's new?**

- Premenopausal women with type 1 diabetes describe their sexual experiences and challenges in the presence of type 1 diabetes.
- Female sexual function should be discussed in diabetes care and management.
- Data from this study will be used to develop a tool to measure sexual function in women with type 1 diabetes.

## 1 INTRODUCTION

Sexual activity and relationships are important dimensions of quality of life.^1^ Problems related to sexual function have been well documented although the extent of the issues are yet to be fully understood.^2^ Prevalence studies differ in how sexual dysfunction (SD) is defined and measured, leading to conflicting findings affecting research outcomes.^2^ This is more apparent in the case of female SD (FSD) as the parameters and associated risk factors are less well defined compared to male SD.^3^ The World Health Organization (WHO) define SD as “syndromes that comprise the various ways in which adult people may have difficulty experiencing personally satisfying, non‐coercive sexual activities”, and refer to sexual response as “a complex interaction of psychological, interpersonal, social, cultural and physiological processes and one or more of these factors may affect any stage of the sexual response”.^4^ Chronic conditions such as diabetes have been linked to impaired sexual function in both males and females, and studies have suggested that the prevalence of SD is higher in women with type 1 diabetes compared to women with type 2 diabetes and women without diabetes.^5^, ^6^ In a review we conducted, the prevalence of SD in women with type 1 diabetes was three fold higher compared to women without diabetes (odds ratio = 3.8 95% CI 1.8‐8.0, *p* < 0.001).^6^ There are multiple psychosocial factors that can impact on sexual function in women with type 1 diabetes, these include depression; diabetes distress; and altered body image.^7^, ^8^ These factors can be compounded, inhibiting women's engagement in and enjoyment of sexual interactions. Diabetes technologies (insulin delivery methods and sensors) may also mediate SD.^8^, ^9^ This impact can be both negative and positive.^10^, ^11^ However, there are no diabetes‐specific measures to address SD in women with diabetes. In our previous systematic review examining the prevalence of SD among women diagnosed with type 1 diabetes,^6^ the predominant assessment tool used was the Female Sexual Function Index scale (FSFI) developed by Rosen et al.^12^ The FSFI measure is based on the International Classification of Diseases (ICD‐10)^13^ and the Diagnostic and Statistical Manual‐IV (DSM‐IV) diagnostic criteria for SD.^14^ The FSFI scale was developed to assess six domains of the female sexual response phases including sexual desire, sexual arousal, orgasm, lubrication, satisfaction and pain. Although these domains address important dimensions of the sexual response phases, they do not address diabetes‐related factors, such as diabetes distress and hypoglycaemia, that may contribute to FSD. For example, a reduced sexual desire due to fear of hypoglycaemia is unique to women with diabetes and would require a different approach to a general loss of desire.

The aetiology of FSD in women with type 1 diabetes is multifactorial. Hyperglycaemia is the main physiological driver causing neurovascular damage, hormonal changes and recurrent genitourinary infections.^7^ Hence, the experience of FSD in women with type 1 diabetes might therefore be distinct from women without diabetes and involves an interplay between many factors. However, despite the high prevalence of SD in women with type 1 diabetes and its negative impact on women's quality of life and mental well‐being, the issue of SD remains underreported in women with diabetes.^15^ There is a general scarcity of research on SD in women with diabetes, especially studies of women's experiences of SD and how diabetes impacts on those experiences.^8^ This paper reports the findings of a study detailing the perspectives and experiences of women with type 1 diabetes in sexual relationships and interactions. The study was conducted as part of a wider project, which will develop a patient‐reported outcome measure (PROM) of FSD specifically for women with type 1 diabetes. The study sought to build on current knowledge and measures of FSD, including the FSFI scale and with reference to the WHO definition of SD.^4^

Our work follows the COnsensus‐based Standards for the selection of health Measurement INstruments (COSMIN) criteria for evaluating the quality of PROM.^16^ A key first step in the COSMIN process is to use exploratory research to explicate the concepts and constructs related to the phenomenon of interest (i.e. FSD in type 1 diabetes) to provide a framework for the measure and to help construct the key questions to include within it. This study is related to that first step.

## 2 METHOD

The aim of the study was to explore women with type 1 diabetes' experiences of SD to develop a thematic framework of those experiences. The thematic framework will be used to inform the domains and the constructs to be included in a new diabetes‐specific measure for FSD. The study objectives were to:

- Explore the experiences of SD for women with type 1 diabetes in the context of their diabetes.
- Identify diabetes‐related factors that impact on women's sexual interactions, behaviours and emotions.
- Characterise how diabetes‐related factors contribute to the experiences of SD in women with type 1 diabetes.

The study used semi‐structured interviews to explore the experiences and views of women with type 1 diabetes in relation to SD. Data were analysed using Framework Analysis (FA)^17^ to identify the key concepts for measuring FSD in the context of type 1 diabetes. National Health Services (NHS) ethical approval was received from the Health Research Authority/Health and Care research/Wales (REC 22/SW/0092, dated 08/07/2022) to conduct interviews with NHS participants, and from King's College London (HR/DP‐22/23–36,186 dated 16/06/2023) to conduct interviews with online participants.

The reporting of the qualitative method was checked against the COREQ (Consolidated criteria for Reporting Qualitative research) checklist,^18^ (see Data S2).

### 2.1 Recruitment and setting

The women were recruited from two NHS diabetes centres in England (University Hospital Bristol and Weston NHS Foundation Trust) and also through social media platforms (Facebook, X (formerly Twitter), Instagram). Online recruitment was included to maximise the heterogeneity of participants to enable a fuller understanding of SD for women from a wide range of backgrounds.

National Health Services participants were recruited from diabetes clinics in the participating centres via a poster advertising the study. In addition, the researcher approached women with type 1 diabetes <50 years of age attending the clinic or from the clinic registry. The study was explained to potential participants in a sensitive and factual way, highlighting that all women in this age bracket were being asked so that the women did not feel embarrassed or that they were being selected for some unknown reason. Women who showed interest in the study were then screened for eligibility verbally in face‐to‐face interviews and via telephone conversation or an e mail in virtual interviews using three screening questions:

‐ Do you experience any anxiety or difficulty in respect to the intimate relationship with a sexual partner?
‐ Do you find it difficult to enjoy sexual relationships?
‐ Do you find intercourse unpleasant or uncomfortable in any way?

Women who answered positively to one of these questions were eligible to be interviewed and those who wished to‐do‐so went through the consenting process following the study ethics. Recruitment for online participants was undertaken via social media platforms. An e‐poster advertising the study and the researcher's contact details was posted online in type 1 online women's Facebook groups or by tagging diabetes organisations in posts on X and Instagram.

The face‐to‐face interviews were conducted in a clinic room within the outpatient department of one of the participating sites, and the virtual interviews were conducted via Microsoft Teams.

### 2.2 Participants and sampling

The study included women (self‐defined) with type 1 diabetes for more than 1 year and who are:

- Sexually active (regularly involved in a sexual act with another person(s))
- Aged 18–50 years
- Able to communicate verbally and in writing in English
- Able to provide an informed consent
- Experiencing sexual difficulty based on their answers to the screening questions

The reason for excluding women who aged above 50 years was to avoid confounding factors related to the menopause, which is also known to influence women's sexual experiences.^19^ The median age of menopause in women with type 1 diabetes is 50 years.^20^ Hence, 50 years of age was used as the criteria for defining menopause.

Participants who did not meet the inclusion criteria or answered negatively to the screening questions were excluded. Women were recruited using purposive sampling based on the study inclusion criteria. The proposed sample size was 20, following the model of information power outlined by Malterud et al.^21^

### 2.3 Data collection

Data were collected using semi‐structured individual interviews^22^ conducted online via Microsoft Teams or in person in a quiet venue within the outpatient department in Weston hospital, depending on preference. The interview topic guide (see Data S1, Table S1) was developed by the research team based on existing literature and reflecting the study objectives. Open‐ended questions were used to elicit in‐depth narratives of the women experiences. The interviews lasted between 20 and 40 min and were conducted by the lead researcher (R.H.), who is experienced in interviewing. The audio recordings of the interviews were pseudo‐anonymised and transcribed verbatim by a transcription company and via Microsoft Teams. Prior to each interview, participants completed a demographic questionnaire to capture data related to their age, age at onset, duration of diabetes, last known glycated haemoglobin (HbA1c), body mass index, use of glucose sensor, method of insulin delivery, relationship status and parity.

### 2.4 Data analysis

Data were analysed thematically using FA.^17^ FA involves minimal data transformation meaning that the researcher is able to stay close to raw data as reported by the participants.^23^ Hence, the themes and subthemes were derived inductively from the data. FA also involves a deductive process to integrate prior theory into the analytical framework, allowing the analysis to build on established theoretical constructs. In this study, we integrated the themes with existing data and theory for SD, to create a framework of definitions for the domains of measurement to be included in the PROM and to inform item generation.^24^

Transcribed data were checked for accuracy against the recording and imported to NVivo, version 14, for analysis. The data were analysed by R.H. with members of the research team verifying the codes and themes. Disagreements were resolved through discussion. The analysis took the following approach: (1) familiarisation—each transcript was read and reread; (2) identification—an initial coding structure was constructed reflecting the interview questions and study objectives; (3) indexing—the initial coding structure was applied to the transcriptions and was adapted based on the content of the interviews and organised thematically; (4) charting—the indexed data were checked to test the fit of each code to the subthemes and themes, and necessary adaptions were made (this was performed by members of the research team, R.H., R.F., J.P., A.F.); (5) mapping—the data were grouped based on areas of convergence in the data to different facets of FSD such as enjoyment or engagement. A reflexivity statement is provided in Data S3.

## 3 FINDINGS

Overall, 18 women with type 1 diabetes participated in the study. Out of the 46 women contacted from the NHS sites, 13 participated. The remainder either did not meet the inclusion criteria (*n* = 8), declined to participate (*n* = 8), or did not respond despite having agreed to receive information about the study (*n* = 17). Five further women were recruited from social media platforms. The participants ages ranged from 22 to 49 years with a median age of 33 years. Duration of diabetes and HbA1c ranged from 2 to 40 years and 37–91 mmol/mol (5.5%–10.5%), respectively. Most of the women were white (*n* = 16), just over half were in a relationship (*n* = 10) and using multiple daily injections of insulin (*n* = 11), the remainder had an insulin pump (*n* = 7). All women used a glucose sensor device (see Table 1).

**TABLE 1:** Characteristics of study participants.

Participant	Age	Age of DM onset (years)	Duration DM (years)	HbA1c mmol/mol (%)	BMI	Glucose sensor (yes/no)	Relationship status (yes/no)	Parity	Insulin delivery
01	37	21	16	51 (6.8)	32	Y	Y	0/0	MDI
02	41	5	36	91 (10.6)	38	Y	Y	2/2	MDI
03	38	36	2	55 (7.2)	28	Y	N	0/0	MDI
04	44	3.5	40	78 (9.3)	28	Y	‐	1/1	MDI
05	26	16	10	‐	27	Y	N	0/0	MDI
06	43	20	23	‐	22	Y	Y	2/0	Insulin pump
07	25	15	10	‐	21	Y	Y	1/1	MDI
08	31	7	24	47 (6.5)	27	‐	Y	0/0	Insulin pump
09	31	17	14	72 (8.7)	32	Y	Y	2/2	Insulin pump
10	32	2	30	‐	21	Y	N	0/0	Insulin pump
11	32	13	19	70 (8.6)	23	Y	N	0/0	Insulin pump
12	34	14	20	58 (7.5)	26	Y	N	0/0	MDI
13	22	14	8	39 (5.7)	22	Y	N	0/0	MDI
14	49	32	18	46 (6.4)	28	Y	Y	2/2	Insulin pump
15	36	34	2	43 (6.1)	23	Y	Y	1	MDI
16	23	16	7	47 (6.5)	‐	Y	N	0	MDI
17	29	25	4	48 (6.5)	23	Y	N	0	MDI
18	29	9	20	37 (5.5)	‐	Y	Y	1	Insulin pump

*Note*: Parity: pregnancies/live birth.

Abbreviations: BMI, body mass index; DM, diabetes mellitus; MDI, multiple daily injection.

### 3.1 Themes related to women's experience of SD

The analysis identified five themes which were related to different elements of FSD. These are: initiation of sexual activity, sexual confidence, sexual enjoyment, sexual engagement and sexual desire. The identified themes and subthemes are presented below with data excerpts reflecting the range of experiences and views expressed by the women and are summarised in Table 2.

**TABLE 2:** Themes related to women experience of sexual dysfunction (SD).

Subtheme (ST) and definition	Data excerpts (participant identification number and age)
(a) Theme 1: Initiation of sexual activity	
*ST*: Fears *Definition*: Concerns about changes in glucose levels and how to deal with the consequences of this	Fear of hypoglycaemia There is a fear there with, you know, having a hypo or sometimes when you have a hypo you just … you're sweating you're shaky, you can't communicate the same. So being with other people and that happening, it's quite … like worrying like it makes me feel quite vulnerable. (13—aged 22 years) If I'm feeling low like I'm not going to be pursuing sex. It's just not gonna happen until like I'm feeling better. (08—aged 31 years) Erm I usually check first before we have sex, just to see if I'm heading up or down. Erm so it doesn't really interfere—I suppose actually if I'm heading low then we don't have sex or at least wait to see if we can have sex later, if we're still awake. (06—aged 43 years) Impact of glucose levels You can't ever let go 100%, you've always got to think, ah is that going to affect my sugar levels … erratic sugar levels, erm, play a part, because if my sugar level's low I can be very emotional and if they go high I can be very irritable, so emotionally that has an affect because I don't particularly err, want to be with my husband. (02—aged 41 years) Yeah, and again when you're running high, it's just, that's the last think you feel like doing [Laughs]. All you want to do is just sit quiet with a very large glass of water and just try and sort your you know [blood sugar] err dehydration from the high sugar levels, I get that very badly. (09—aged 31 years)
*ST*: Body image impact of diabetes *Definition*: Concerns and fears related to diabetes body image issues and initiating of sexual activity, such as injection sites, diabetes technology and other physical effects of diabetes	Injection sites I've injected for so long, I have little pockets of what looks like fat on the tops of my arms, the tops of my thighs, my tummy, and so all the areas where you inject. So it—it makes me self‐conscious of my body, which obviously, when you're being intimate, is—I'm self‐conscious of that. Which can have a negative effect on everything. (10—aged 32 years) I lost my virginity when I was nearly 19, one of the things I was concerned about I've got pockets from my injections on my stomach and I've got like the size of my hand a huge cyst in my right leg and I have stretch marks from my injection sites, I used to get really nervous about just showing those and getting asked questions when I was doing my injections, It is more of an emotional psychological thing rather than a physical discomfort. (12—aged 34 years) Physical effects of diabetes I am I'm aware of hygiene. So I'm very concerned about oral sex in terms of how clean I am because I'm aware of the smell of the urine of a person with diabetes. (14—aged 49 years)
*ST*: Internalised stigma *Definition*: The impact of internalised stigma on initiating sexual activity	Acceptance of diabetes With new people, it's never … I just don't want to bring it up as a thing that I have. Um, as a disability, I think. Because people see it … it is a disability, it's not something you'd want to come out with straight away. Um, or maybe … I don't know. I'm not comfortable with one of the first things I say is me saying that I'm diabetic and I have a disability. So I just, sort of, wait until they're a friend, I guess, to at least tell them, maybe. (05—aged 26 years)

(b) Theme 2: Sexual confidence	
*ST*: Body confidence *Definition*: How diabetes affects their self‐esteem and bodily confidence either generally or in relation to the impact of insulin (lipodystrophy)	Body image I don't want to blame diabetes for me not having the best self‐esteem, but I do think it's a bit of a challenge to feel as beautiful about your body as somebody who doesn't have all the stuff attached to them or marks or injection sites or finger pricking or having to test your sugar before or after sex or any of that sort of thing that comes along. (14—aged 49 years) I've got lumps and bumps all over the place, erm, and I think after having it for so long it's inevitable that you're going to get some like hypertrophy so erm which again makes you feel really unattractive. (02—aged 41 years) Body confidence But I think for me it is also related to erm like lack of self‐love and self‐esteem. Erm I don't think I felt very confident in my body. So like independent of my diabetes. So I kind of felt like this was another thing that would kind of decrease my attractiveness to somebody else. (08—Aged 31 years) It is being self‐conscious of my body, and there's—it's kind of like I feel more comfortable with the lights off now [laughs], or at least dimmed. Whereas I never used to be conscious of that. (04—aged 44 years)
*ST*: Impact of visible diabetes technological devices *Definition*: Visible diabetes devices can affect how women feel about their bodies and their confidence to engage in sexual activity. Women can also be anxious that the device may become dislodged or damaged during sexual activity, impacting on their confidence	Technology and body image I've just always felt like a little bit nervous and insecure about just having my like infusion set on display erm during intimacy. (08—aged 31 years) I've never wanted an insulin pump purely because of how it looks, do you know what I mean? (13—aged 22 years) Like at times when I'm feeling more intimate, only thing ohh you know like I will put it somewhere that's not as noticeable. Um, because it is, it's a bit bionic. (14—aged 49 years) But the reason that I chose the pod rather than the other pumps with the wires is that I really didn't want to have visible tubes sticking out of me because they would make me feel like I looked ill … like people in hospital that have got cannulas … I didn't want that to be how I appear. (06—aged 43 years) So yeah, I think in terms of my intimate life, I was worried that this (diabetes technology) could have an influence on how people would perceive me. (17—aged 29 years) Anxiety about devices If I'm in bed, I'm aware that I can knock it out. I know there's patches and things. And I'm also aware that it's a bit unsightly, because my partner has suggested to me that I buy a patch, or he'll—he will buy me patches to cover it up, and I think, ‘Is that because that it doesn't look nice?’ (04—aged 44 years)

(c) Themes 3: Sexual enjoyment	
*ST*: Physical impact of diabetes symptoms *Definition*: The effects of diabetes on bodily structures involved in sexual activity and intimacy can reduce sexual enjoyment	Genitourinary issues And from a physical point of view, erm, with the sugar levels being all over the place, erm, it seems to have a physical affect as well so sometimes you get dryness, I find when my sugar levels are higher erm, I'm more prone to erm bladder infections, so every time we're together in the back of my mind somewhere there's this, is this going to result in another bladder infection? (02—aged 41 years) There have been periods when I had thrush a lot … so that's affects my sexual activity. (12—aged 34 years) Vaginal dryness and pain I went to various GPs, erm because I also—it was causing quite a lot of pain erm and I was having tears and the skin around the vagina was getting very dry as well. (06—aged 43 years) Oral dryness It's happened a few times where I've gotten intimate with someone and my … the dryness of the mouth affects how I do things or how I'd normally want to do things. And I think it's … it affects the intimacy. (05—aged 26 years) Lack of pleasure I don't feel as satisfied as what I used to. And that's not because of anyone's performance. It's more about me. (04—aged 44 years)
*ST*: Glucose levels *Definition*: Sexual enjoyment is impacted by glucose levels particularly hypoglycaemia	Hypoglycaemia Erm but I can't like—I cannot erm like have an orgasm if I am low. Like it can't happen. It just cannot happen. I find like physiologically it can't happen. (08—aged 31 years) Oh, there's been occasions where I've had to stop, because I'm having a hypo, and it's … that's not very romantic. (04—aged 44 years)
*ST*: Impact of diabetes technology *Definition*: Diabetes technology functions such as glucose alarms can impact on sexual enjoyment	Glucose alarms But it's always worse when we're in the moment and the alarm goes off or something cause he just freezes. It doesn't ruin the moment completely, but it does stop it enough that everyone has a little chat. (18—aged 29 years)

(d) Themes 4: Sexual engagement	
*ST*: Technology disruption *Definition*: Concerns that diabetes technology such as glucose sensors may be impacted	Worry about diabetes devices. So I think the biggest one that comes to my mind was the anxiety that my devices would be ripped off through intercourse and that, yeah, that was like from the first day. And when I was using this technology, I was very worried that, you know, something might happen to the sensor or Dexcom that it will like, you know, destroy the atmosphere of the moment. (17—aged 29 years) The pump definitely has an affect because you're constantly worried about where it is, I mean, I know from previous sexual activities before it has fell out. (09—aged 31 years) I was definitely wanting to be more sexually active than we were, but because of how much the cannulas were leaking, I know I was over tired mentally. I was feeling drained from a diabetes perspective and I was just wanting to sleep. (18—aged 29) There have obviously been a few occasions where the—my pump has been ripped off due to like movement or the like rigour I guess of, of having sexual activity … then we have to stop and then like—I have to do the whole insulin thing again. (10—aged 32 years)
*ST*: Impact of glucose levels on sexual engagement *Definition*: The impact of glucose levels, particularly hypoglycaemia, in relation to sexual engagement and activity	Hypoglycaemia It just takes the spontaneity out of it though, err [Laughs] erm, … so when I have had hypos we've had to stop and then obviously there's my husband's side of it which is obviously he's not got what he's wanted and there's perhaps disappointment there. (02—aged 41 years) The issue I have with hypos is that I'm very self‐conscious, because I'll start sweating, and I can be in bed with someone, and it's kind of like—oh, it's not—even if we're just asleep, it's just being close to someone and having a hypo, just—yeah, it just stresses me out. (04—aged 44 years) It could be like during an intimate moment … I almost have to like disrupt that and just be like, ‘Can I just—I'm, I'm just going to test really quickly and see where I'm at because I'm not feeling great?’ (10—aged 32 years)
*ST*: Physical complications of diabetes *Definition*: Genitourinary complications of diabetes can impact sexual engagement and add additional considerations that may complicate sexual engagement	Dryness Is it because I need to be using more lubrication or you know because my sugars are probably a little bit higher than they should be, so all of the above I think have a massive impact on engagement. (14—aged 49 years) Risk of urogenital infection I worry because I am diabetic, I catch everything that goes around. And I am really conscious of making guys wear condoms and maybe they don't always want to wear a condom. (12—aged 34 years) Also, obviously due to having diabetes, sometimes I can get thrush affecting my sex life. (13—aged 22 years)

(e) Themes 5: Sexual desire	
*ST*: Impact of glucose levels *Definitio*n: High, low and fluctuating glucose level can reduce sexual desire by lowering mood and energy	Feeling tired But if you've had a bad day with blood sugars or you've been high, then I would definitely feel tired if I've had a bad night. I'm just not the same and I know that when I was running my blood sugars lower a lot of the time. I just felt tired so wasn't really up for it, but by my glucose management I definitely say if you're less stable then your desire just isn't really there. (18—aged 29 years) Impact on mood Oftentimes during periods of swinging glucose and high variability, you are not in the mood to have sex because your blood glucose is all over the place. And I think that plays a huge role in intimacy. (07—aged 25 years)
*ST*: Reduced desire *Definition*: A perceived decline in desire possibly linked to diabetes complications	Diminished desire I don't know whether it affects my desire for wanting to have sex, but that seems to have diminished, maybe, where—as complications, diabetic complications have arose. I don't know whether that's linked, myself, but I have noticed that the yearning's not there for me. (04—aged 44 years) I feel satisfied, but that's when it happens because I just don't, like I said, I just don't have the desire to, not for somebody of my age. (09—aged 31 years) Partner's pleasure You don't necessarily want to be as physical as you once were … sometimes it feels like I kind of have to push myself to perform a little bit because erm, I want them (partner) to enjoy it even if I'm not enjoying it myself. (02—aged 41 years) Worry about lack of desire It's been a worry for me, because I kind of thought that women in their 40s were at their peak, and I feel like I'm not, and it makes me think that there's something wrong with me, and I don't know. (04—aged 44 years) Avoidance of sexual activity There's no desire there, and it's not because I'm not attracted to my partner, it's just the way I feel, and I kind of I'm trying to avoid it, and I think it's become apparent that I'm trying to avoid it, which is causing a few issues. (02—aged 41 years)

#### 3.1.1 Theme 1: Initiation of sexual activity

Initiation of sexual activity refers to the ability of women with type 1 diabetes to communicate their sexual interest both verbally and nonverbally. The women talked about issues that impacted their ability to initiate sexual activity, both with long‐term and new partners. This theme included three subthemes highlighting factors associated with reduced initiation of sexual activity, these were:

1. Fears—Women relayed how they experienced fears in initiating sexual activity due to their glucose levels, whether it was hypoglycaemia or hyperglycaemia. Uncertainty about their glucose levels seemed to drive women away from their sexual partners affecting their ability to initiate sexual activity.
2. Body image impact of diabetes—Some of the women reported how diabetes‐related changes to their bodies inhibited their confidence in initiating sexual activity. Women described how having pockets of fat from insulin injections (lipohypertrophy) made them feel self‐conscious about their bodies.
3. Internalised stigma—Women were fearful that they might be judged by a new partner because of their diabetes leading them to avoid initiating sexual activity. (see Table 2a).

#### 3.1.2 Theme 2: Sexual confidence

Female sexual confidence was defined as having the confidence and ability to express sexual interest and being comfortable within one's own body in the presence of challenges related to living with type 1 diabetes. The women reported a range of issues related to diabetes that influenced their confidence in expressing sexual interest. Similar to the first theme, these arose from body image issues related to diabetes. Two subthemes were identified related to the physical impact of diabetes on their bodies and the use of visible diabetes technologies.

1. Body confidence—Bodily changes related to insulin use (lipohypertrophy and insulin disruptions) led women to feel unattractive impacting self‐esteem and reducing their sexual confidence.
2. The impact of visible diabetes technological devices—Diabetes technologies (sensors and insulin pumps plus associated paraphernalia) impacted negatively on sexual confidence, feeling nervous the technology may get dislodge and insecure about how the “bionic” devices are perceived by others. (see Table 2b).

#### 3.1.3 Theme 3: Sexual enjoyment

Sexual enjoyment was defined as the physical and/or psychological pleasure experienced during sexual activity. Sexual pleasure experienced by women as a result of sexual activity was negatively impacted by diabetes‐related concerns such as diabetes‐related genitourinary infections and hypoglycaemia. Three subthemes covering physical and psychological factors which impact on sexual enjoyment were identified.

1. The physical impact of diabetes symptoms—Women who experienced vaginal or recurrent bladder infections, which they related to their diabetes, felt that these problems negatively impacted on their sexual pleasure.
2. Glucose levels—Hypoglycaemia either before or after sexual activity was reported to impact on women's ability to reach orgasm or to fully enjoy sexual activity.
3. Impact of diabetes technology—This related particularly to glucose sensors incorporated alarms. The alarms can disrupt sexual enjoyment for both women and their partners (see Table 2c).

#### 3.1.4 Theme 4: Sexual engagement

Sexual engagement in this study refers to the act of being physically involved in a sexual activity. Women experienced a lack of engagement resulting from emotional and physical diabetes‐related factors, these are expressed in three subthemes.

1. Technology disruption—Women can experience problems with diabetes technologies during sexual activity, particularly associated with devices dislodging during sexual activity leading to cessation of the sexual engagement or cancelling the sexual activity.
2. Impact of glucose levels—Women reported how hypoglycaemia can take the spontaneity out of the sexual experience, and that autonomic symptoms such as sweating can create anxiety and cessation of engagement leading to disappointment for women and their partners.
3. Physical complications of diabetes—Physical problems such as vaginal dryness and anxiety about developing genitourinary infections following intercourse, can negatively impact sexual engagements (see Table 2d).

#### 3.1.5 Theme 5: Sexual desire

Sexual desire describes the willingness to initiate and/or take part in a sexual activity. The women talked about changes in their level of desire, some of which they related to their diabetes although there was also a more general lack of desire that they could not directly attribute to their diabetes. This theme is comprised of two subthemes:

1. Impact of glucose levels—Women reported that high, low or variable glucose levels reduced their desire for sexual activity as it led to tiredness or reduced motivation.
2. Reduced desire—Women reported general lack of desire which seemed to develop as they also acquire diabetes complications, although the women could not directly associate them. Some women said that this meant they only engaged in sexual activities for their partner's pleasure rather than their own (see Table 2e).

**FIGURE 1:**
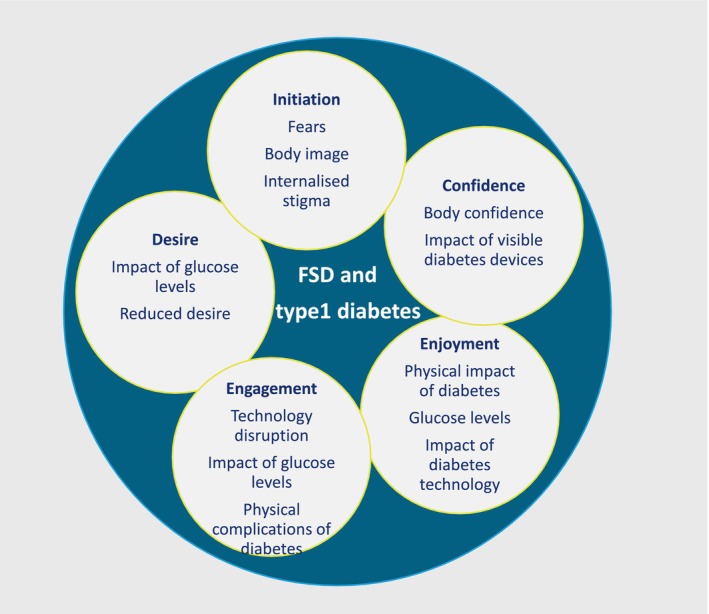
Female sexual dysfunction (FSD) and type 1 diabetes main themes.

The five themes are presented schematically in Figure 1. The figure shows how there was an overlap between the factors identified in relation to each theme, particularly glucose levels and diabetes technologies.

#### 3.1.6 Domain definitions

In the final step of the analysis, the themes were integrated with existing SD theory to inform domain definitions for PROM development. These domains will provide a framework of measurement and item generation for the PROM based on the experiences captured in this study (see Data S2, Table S2).

## 4 DISCUSSION

This study revealed that FSD in the presence of type 1 diabetes is a complex phenomenon resulting from multiple interacting factors that can influence one another. The study presents a novel insight into the challenges of SD as described by the women themselves providing a framework for identifying PROM for the assessment of FSD. The women talked about the impact of diabetes on their experiences of sexual activities. In the context of this study, the term sexual activity refers to a broad range of intimate behaviours and processes,^25^ such as intercourse, touching and close companionship, between two persons involving sexual arousing.^26^

Issues around glucose activities in relation to hypoglycaemia and hyperglycaemia were regularly reported by the women. Hypoglycaemia is known to impact the level of sexual activity in people with diabetes.^27^ Previous studies have reported positive correlations between elevated HbA1c levels and FSD highlighting the association between FSD and hyperglycaemia.^28^ Furthermore, glucose variability, rather than long exposure to hyperglycaemia, was found to cause a threefold increase in risk of FSD.^29^ This would suggest that factors impacting glucose levels leading to glucose activity need be addressed in the assessment of FSD in women with diabetes.

The women reported issues related to vaginal and oral dryness, pain and problems with orgasm and this affected their ability to initiate, engage in or enjoy sexual activity. Similar issues were reported in a previous qualitative study by Buskoven et al.^8^ While the underlying causes of these problems were generally detailed in relation to hyperglycaemia in that study, they also highlighted that psychological challenges and the demands of diabetes exacerbated the impact of hyperglycaemia on the sexual experiences of women with type 1 diabetes.

The women experienced negative feelings related to body image stemming from injection marks and visible diabetes technological devices. Other studies have also reported that negative body image concerns are associated with fear and avoidance of physical intimacy, and it can affect the frequency and quality of sexual activities.^30^ However, the impact of diabetes technology on women's sexual function is not fully understood as current evidence is inconsistent. Some studies report a positive impact associated with the use of diabetes technological devices due to improvement in the prevention of hypoglycaemia and in quality of life.^30^, ^31^ While others have reported that the use of insulin pumps caused difficulty during sexual intercourse due to bodily discomfort.^27^ A survey of 289 women with diabetes found that 50% of insulin pump users reported insulin pumps interfering with sex, and 75% disconnected their pump during sexual activity.^9^ The same study also found that 20% of continuous glucose monitoring (CGM) users found that CGM interfered with sexual activity. This highlights the importance of working with the women towards a treatment option that reduces body image distress or improving women's confidence to manage these technologies in the context of intimacy. Given the increased use of diabetes technology and its potential impact on sexual function raises the question of whether SD should be discussed more routinely in diabetes clinics.

The women reported sexual relationship issues with long‐term and new partners as a result of diabetes. They reflected on the importance of having a diabetes‐supportive partner, who was described in their words as a person who is understanding empathetic and has knowledge about their condition. The impact of diabetes on relationships (and vice versa) has been identified as an important social mediator of women with type 1 diabetes' quality of life.^31^ A study by Helgeson et al.^32^ found that people with diabetes believe that diabetes affects both individuals in a relationship and that it was a source of distress to their partners, and this affected their ability to express love. Therefore, resources or education on diabetes in relationships should be available for both people with diabetes and their partners.

### 4.1 Implications

This study has highlighted that SD is experienced in multiple ways and is related to a wide range of issues for women with type 1 diabetes, with significant impact on their intimate relationships. The findings of the study were used as a framework for the development of a new PROM for assessing SD in women with type 1 diabetes, which we are currently testing. Following the COSMIN guidance which emphasises the importance of acknowledging peoples' experience of their conditions,^16^ we have used the findings to generate an item bank for the new questionnaire. The items have been framed reflecting the phrasing used by women in the quotes with the intention that it will resonate with the women by using a similar language as much as possible.^24^ The development of diabetes‐specific PROM for FSD will hopefully help to increase the visibility of the problem and in establishing treatment pathways that address the potential impact of diabetes and its associated technologies on women's experiences of intimacy and sexual interactions.

### 4.2 Study limitations

While this study has identified a range of different diabetes‐specific factors that may mediate the experience of SD for women with type 1 diabetes, it is important to consider these findings in relation to some of the limitations of the study. Firstly, all but one of the women were in heterosexual relationship, precluding any exploration of SD in same‐sex relationships. Hence, further research is needed to explore SD in the lives of women with type 1 diabetes in same‐sex relationships. While the study was promoted to all women with type 1 diabetes in the recruitment contexts, it is possible that to explore SD in same‐sex relationships would require a more explicit or focussed approach. In addition, only two non‐white women participated in the study limiting any exploration of SD in women of colour. It was the intention to recruit more women of colour online as the populations of women in the two participating diabetes centres were predominantly white, but this did not manifest. While recruiting people from ethnic minorities in research is known to be challenging, it is important, particularly in research that will inform a PROM development, to include participants reflective of the population. Again, this suggests a more targeted approach may help improve recruitment from diverse populations.

Secondly, the study recruited women from the UK only, limiting the opportunities of capturing financial and cultural factors that may impact sexual function in women with type 1 diabetes in other countries including low‐ and middle‐income countries. Hence, it would be interesting to conduct similar research in other countries, although as the findings in our study relate to those reported previously from other contexts it is likely that what we have found would be relevant in other national populations. We will also seek to validate the FSD PROM we are developing in other populations.

Thirdly, it is important to acknowledge that SD affects women of all ages including those in the peri and postmenopausal phases. The experience of SD in postmenopausal women with type 1 diabetes may be more distressing with the addition of factors related to aging. The reason that this study focused on premenopausal women was to enable a focus exclusively on the impact of diabetes on FSD independent of the more generic age‐related factors associated with menopause. While this focus appears to have been successful in explicating the multiple diabetes factors that associate with SD, exploring these factors with older women and indeed young women under the age of 18 years should be considered in future studies.

### 4.3 Conclusion

The experiences of SD in women with type 1 diabetes are related to multiple factors specific to diabetes, including diabetes treatments and technologies. Blood glucose regulation including both hyper and hypoglycaemia impact on women's engagement in and enjoyment of sexual intimacy. Given the strong relationship between sexual function and diabetes, there needs to be much greater consideration of FSD in diabetes care and management.

## AUTHOR CONTRIBUTIONS

RH collected and analysed the data. AF, RF and DA contributed to the data analysis. RH wrote manuscript. AF, RF, DA and JP reviewed and edited manuscript.

## FUNDING INFORMATION

This work was supported by PhD fellowship funded by the Foundation of European Nurses in Diabetes and a grant from Novo Nordisk UK Research Foundation.

## CONFLICT OF INTEREST STATEMENT

The authors declare that they have no known competing financial interests or personal relationships that could have appeared to influence the work reported in this paper.

## Supporting information


Data S1.

Data S2.

Data S3.

## Data Availability

The data that supports the findings of this study are available in the supplementary material of this article.
