# Supplementary material for: Qualitative study exploring the experiences of sexual dysfunction in premenopausal women with type 1 diabetes
Source: Diabet Med. 2024 Sep 20;42(1):e15439. doi: 10.1111/dme.15439 (PMC11635588; doi:10.1111/dme.15439)
Supplement: Supplementary file 1 — Data S1. [file DME-42-e15439-s002.docx]

**Table 1: Interview guide**

| **Main questions** | **Additional questions** |
| --- | --- |
| **1: Thinking about your intimate relationships with others, tell me about any issues/ problems/ difficulties you have experienced due to your diabetes** | Do you think that having diabetes has had an impact on having and sustaining intimate relationships?  Thinking about your:   - Blood glucose levels - Worries about hypos - Any diabetes technologies you use   How do you feel about it?  Wearing visible diabetes technology how does it make you feel… what if any influence does it have on how you feel about your body…makes you less or more attractive? |
| **2: When were you diagnosed with diabetes? If in adulthood, what (if anything) is different about your experiences of being intimate since your diagnosis?** | Has having diabetes had an impact on you in terms of initiating relationships with new partners?  What has been your experiences of how partners respond to your diabetes? |
| **3: Can you tell me whether you experienced any specific difficulty in your sexual intimacy including having problematic relationship?** | If you had diabetes later in life, do you experience SD more since you were diagnosed with diabetes?  What impact has your DM diagnosis had on this? |
| **4: Have you had any support in respect to any sexual problem that you may have encountered?** | Can you tell me if anything has been helpful? What type of support did you have if any? What are your thoughts about introducing the issue of SD in your follow up appointment? How did you get the support? If a HCP provided support, who do you think should initiate a conversation about SD (HCPs or PWD) |
| **5: What are your experiences of hypoglycaemia during sexual intercourse?** | Do you try to avoid hypos?  What strategies do you use? Does it work? |
| **6: What are your thoughts about the effect of sexual intercourse on your glucose levels?** | Do you ever think about your glucose levels in relation to engaging in sexual intercourse? |
| **7: Tell me the about the impact of your mental wellbeing on your sexual function if there is any?** | Do you feel depressed because of your diabetes?  Do you feel shame? |
| **8: Do you think that diabetes affected your sexual engagement and satisfaction? In which way?** | Do you reach satisfaction during sexual intercourse?  Do you find intercourse painful? |

**Table 2: Domain definitions**

| **Domain** | **Definition** |
| --- | --- |
| **Initiation of sexual activity** | **Initiation of a sexual activity** refers to the verbal and nonverbal behaviours used to communicate sexual interest (1). Based on the experiences of women represented in the data from this study sexual initiation is defined as: *the initiation of sexual activity and the ability of women with type 1 diabetes to communicate their sexual interest both verbally and nonverbally.* |
| **Sexual confidence** | **Sexual confidence** has been defined in terms of being comfortable to experience sexual desires and having trust in the ability to engage in a sexual activity (3,4). Celik (3) described sexual self-confidence as a phenomenon that can result from self-satisfaction and self-esteem, and it usually correlates with thoughts about body image. People with inadequate levels of self-confidence tends to avoid contact and postpone their sexual desires (3).  Based on the experiences of women represented in the data from this study sexual confidence is defined as: *having the confidence and ability to express sexual interest and being comfortable within one’s own body in the presence of challenges related to living with type 1 diabetes.* |
| **Sexual enjoyment** | **Sexual enjoyment** is impacted by multiple psychosocial and physical factors (4). Type 1 diabetes introduces additional factors that can influence a woman’s ability to enjoy sexual activities. Based on the experiences of women represented in the data from this study sexual enjoyment is defined as:  *the physical and/or psychological pleasure experienced by women with type 1 diabetes during sexual activity.* |
| **Sexual engagement** | **Sexual engagement** can be driven by multiple conscious or subjective drivers. These can be internal; love, the desire to procreate, or for pleasure (6), or external related to relationships, such as pleasing their partners (7). The ability for women with type 1 diabetes to engage in sexual activity can be impacted by multiple psychosocial and physical factors related to type 1.  Based on the experiences of women represented in the data from this study sexual engagement is defined as: *being physically and psychologically confident to engage in a sexual activity taking into account challenges of type 1 diabetes.* |
| **Sexual desire** | **Sexual desire** refers to specific feelings stimulating the individual to look for a sexual experience or as sexual thoughts and a biological urge to initiate a sexual activity (7, 8) Sexual desire can be affected by biopsychosocial challenges of type 1 diabetes. Based on the experiences of women represented in the data from this study sexual desire is defined as: *the willingness of women with type 1 diabetes to initiate and/or take part in a sexual activity.* |
| **Note**  Sexual activity: Refers to broad range of intimate behaviours and processes (9), such as intercourse, touching and close companionship, between two persons involving sexual arousing (10). | |

**References:**

1. Vannier, S.A., O’Sullivan, L.F. Communicating Interest in Sex: Verbal and Nonverbal Initiation of Sexual Activity in Young Adults’ Romantic Dating Relationships. *Arch Sex Behav* **40**, 961–969 (2011). <https://doi.org/10.1007/s10508-010-9663-7>
2. O'Sullivan, L.F. and Byers, E.S., 1992. *College students’ incorporation of initiator and restrictor roles in sexual dating interactions.*
3. Çelik, E. Development of a Sexual Self-Confidence Scale and Its Psychometric Properties. *Hacettepe Üniversitesi Eğitim Fakültesi Dergisi (H. U. Journal of Education)* 2015; 30(1): 48-61 [Ocak 2015]
4. Simsek, N., Evli, M., Uzdil, N. *et al.* Body Image and Sexual Self-confidence in Patients with Chronic Urticaria. *Sex Disabil* **38**, 147–159 (2020). <https://doi.org/10.1007/s11195-019-09610-6>
5. Pauls, FR. Anatomy and the clitoris and the female sexual response. *Clinical anatomy*. 2015; Vol 28 (3).
6. Meston, C.M., Stanton, A.M. Recent Findings on Women’s Motives for Engaging in Sexual Activity. *Curr Sex Health Rep* **9**, 128–135 (2017). <https://doi.org/10.1007/s11930-017-0114-5>
7. Basson R. The female sexual response: a different model. *J Sex Marital Ther*. 2000;26(1):51–65.
8. Kaplan, H. S. A neglected issue: The sexual side effects of current treatments for breast cancer. *Journal of Sex & Marital Therapy*, 1992; 18, 3-19
9. Freak-Poli, R., Licher, S., Ryan, J., Ikram, M.A. and Tiemeier, H.. Cognitive impairment, sexual activity and physical tenderness in community-dwelling older adults: a cross-sectional exploration. *Gerontology*, 2018; *64*(6), pp.589-602.
10. Chung HS, Kim GH, Shin MH, Park K. Physical Intimacy Is an Important Part of Sexual Activities: Korean Older Adults Study. *Sex Med*. 2020 Dec;8(4):643-649. doi: 10.1016/j.esxm.2020.06.011. Epub 2020 Aug 6. PMID: 32773264; PMCID: PMC7691871.
