# Supplementary material for: Qualitative study exploring the experiences of sexual dysfunction in premenopausal women with type 1 diabetes
Source: Diabet Med. 2024 Sep 20;42(1):e15439. doi: 10.1111/dme.15439 (PMC11635588; doi:10.1111/dme.15439)
Supplement: Supplementary file 3 — Data S3. [file DME-42-e15439-s001.docx]

**Supplementary material 3: Reflexivity**

**Study profiles**

The lead researcher RH is a female clinical academic diabetes nurse working in one of the participating NHS sites, and is a doctoral fellow at King’s College London. RF (female) is a clinical academic diabetes nurse and senior lecturer. JP (female) is a diabetes researcher and lecturer. DA (male) is a professor of diabetes nursing, AF (male) is a clinical academic professor of diabetes nursing.

All interviews were conducted by RH who has prior knowledge and experience of qualitative interviewing having previously conducted a qualitative study and completed a research methods course. All other researchers have expertise in the conduct of studies related to diabetes and womens health using a varity of research designs including qualitative methods.

All researchers contributed to the data analysis, which was largely based on the initial interpretation by RH, and drawing on the original transcripts from the interviews.

**Prior relationship with study participants**

RH had a clinical relationship with two participants prior to the interviews. This relationship could have incluenced their decision to participate; however, they only became aware of the study during the recruitment process. The remainder of the participants were unknown to RH. None of the other authors had prior contact with the participants. There was a level of consistency across the data suggesting that RH’s prior clincal relationship did not influence the findings.

As part of the recruitment process a particiapant information sheet outlining the aim of the study and why the research is being undertaken was given to the participants. This may have provided an opportunity for potential participants to read about the researchers’ interest online or influenced their responses and accounts.
